# Quantification of atopy, lung function and airway hypersensitivity in adults

**DOI:** 10.1186/2045-7022-1-16

**Published:** 2011-12-12

**Authors:** Susana Marinho, Angela Simpson, Paul Marsden, Jacky A Smith, Adnan Custovic

**Affiliations:** 1 The University of Manchester, Manchester Academic Health Science Centre, University Hospital of South Manchester NHS Foundation Trust, Manchester, UK

**Keywords:** IgE, atopy, quantitative assay, lung function, airway hyperresponsiveness

## Abstract

**Background:**

Studies in children have shown that concentration of specific serum IgE (sIgE) and size of skin tests to inhalant allergens better predict wheezing and reduced lung function than the information on presence or absence of atopy. However, very few studies in adults have investigated the relationship of quantitative atopy with lung function and airway hyperresponsiveness (AHR).

**Objective:**

To determine the association between lung function and AHR and quantitative atopy in a large sample of adults from the UK.

**Methods:**

FEV_1_ and FVC (% predicted) were measured using spirometry and airway responsiveness by methacholine challenge (5-breath dosimeter protocol) in 983 subjects (random sample of 800 parents of children enrolled in a population-based birth cohort enriched with 183 patients with physician-diagnosed asthma). Atopic status was assessed by skin prick tests (SPT) and measurement of sIgE (common inhalant allergens). We also measured indoor allergen exposure in subjects' homes.

**Results:**

Spirometry was completed by 792 subjects and 626 underwent methacholine challenge, with 100 (16.0%) having AHR (dose-response slope>25). Using sIgE as a continuous variable in a multiple linear regression analysis, we found that increasing levels of sIgE to mite, cat and dog were significantly associated with lower FEV_1_ (mite p = 0.001, cat p = 0.0001, dog p = 2.95 × 10^-8^). Similar findings were observed when using the size of wheal on skin testing as a continuous variable, with significantly poorer lung function with increasing skin test size (mite p = 8.23 × 10^-8^, cat p = 3.93 × 10^-10^, dog p = 3.03 × 10^-15^, grass p = 2.95 × 10^-9^). The association between quantitative atopy with lung function and AHR remained unchanged when we repeated the analyses amongst subjects defined as sensitised using standard definitions (sIgE>0.35 kUa/l, SPT-3 mm>negative control).

**Conclusions:**

In the studied population, lung function decreased and AHR increased with increasing sIgE levels or SPT wheal diameter to inhalant allergens, suggesting that atopy may not be a dichotomous outcome influencing lung function and AHR.

## Background

The association between reduced lung function and allergen sensitisation (mainly to inhalant allergens) has been clearly documented, both among children[1-7] and adults[8], often in the context of high allergen exposure[1,8]. A similar association has also been demonstrated for increased airway hyperresponsiveness amongst atopic individuals compared to those not sensitised[7-13].

Most of the studies investigating the relationship between allergen sensitisation and lung function or airway hyperresponsiveness (AHR) considered atopy as a simple dichotomous variable, assigning individuals as atopic or non-atopic based on arbitrary and differing cut-off points, either for IgE measurement or skin prick testing. [1-5,8-11]. Similar is the case for the studies reporting on the association between atopy and wheeze or other symptoms of allergic disease[14,15]. Analysing sensitisation quantitatively has been shown to improve the specificity of these tests. For example, the level of specific IgE may predict the likelihood of patients having symptomatic food allergy[16] and the size of the skin prick test wheal can be used in a similar way[17]. We have previously demonstrated similar quantitative relationship between specific serum IgE levels to common inhalant allergens and the presence and persistence of childhood wheezing and reduced lung function[6]. We have also shown a similar association between increasing levels of sIgE or size of skin test wheal to inhalant allergens and the presence of childhood allergic rhinitis[18]. However, very few studies in adults have investigated a quantitative relationship between atopy and lung function. A study in the US has demonstrated that AHR increased significantly amongst adult asthmatics with increasing size of skin test wheals to inhalant allergens[11]. A significant association was also reported amongst non-asthmatic individuals with increasing level of mite specific IgE[12].

We aimed to investigate the associations between the quantification of atopy (using specific IgE levels and the size of skin test wheal to a range of common inhalant allergens) and lung function parameters (FEV_1_, FVC) and AHR in a population of adults with and without asthma, evaluating this in the context of smoking habits and indoor allergen exposure.

## Methods

### Study Population

Detailed phenotyping which included information on symptoms and assessment of lung function, airway reactivity and atopy was carried out amongst parents of children enrolled in a population-based birth cohort study (Manchester Asthma and Allergy Study) [19,20]). We enriched the study population with carefully phenotyped asthmatics fulfilling the following criteria: (1) physician-diagnosed asthma; (2) asthma symptoms (wheeze, cough, chest tightness, or breathlessness) in the previous 12 months; (3) currently using asthma treatment; and (4) no asthma exacerbation or respiratory infection within 4 weeks before the study.[8]. Only subjects of mixed European origin were included in this analysis. The study was approved by the Local Research Ethics Committee. Written informed consent was obtained from all subjects.

### Data sources

#### Symptoms

A validated questionnaire[21] was interviewer-administered to collect information on symptoms, physician-diagnosed illnesses, treatments received, pet ownership and smoking habits.

#### Lung function

FEV_1_ and FVC were assessed using spirometry according to ATS/ERS guidelines[22,23], and expressed as % predicted.

#### Airway responsiveness

assessed by methacholine challenge using the 5-breath dosimeter protocol, as per ATS guidelines[24].

#### Atopy

We performed skin prick tests (SPT) to *D. pteronyssinus*, cat, dog, grass pollen mix, tree pollen mix (Stallergénes, France), and mould mix (Dome-Hollister-Stier, USA). We measured specific serum IgE (sIgE) to *D. pteronyssinus*, cat, dog and grass pollen mix by ImmunoCAP^©^ (Phadia, Uppsala, Sweden).

#### Indoor allergen exposure

We visited homes and collected dust samples from the subjects' bed and the lounge floor by vacuuming 1 m^2^ areas for two minutes in a standardised fashion. Mite (Der p 1), cat (Fel d 1) and dog (Can f 1) allergens were assayed using enzyme-linked immunoassays[25].

### Definition of outcomes and exposures

#### Current asthma

Physician-diagnosed asthma with asthma symptoms and/or use of asthma medication in the last 12 months[26].

#### Airway hyperresponsiveness (AHR)

expressed as methacholine dose-response slope (MDRS)[27,28]. Participants were considered to have AHR if MDRS was >25.

#### Allergic sensitisation as a dichotomous variable

SPT wheal mean diameter (WMD)≥3 mm compared to negative control and/or specific IgE (sIgE)>0.35 kU_A_/l to any allergen.

#### Tobacco smoking

Questionnaire information on smoking habits was used to derive continuous measures of smoke exposure for all subjects as smoke-pack-years (SPY): non-smokers = 0; calculated for both current and ex-smokers (SPY=number of cigarettes smoked per day/20 × number of years of smoking).

#### Allergen exposure

Individual exposure to house dust mite (Der p 1), cat (Fel d 1) and dog (Can f 1) allergens were expressed as allergen concentration per gram of fine dust (μg/g)[29].

### Statistical analysis

The primary outcome measures were lung function parameters and AHR. All dynamic lung volumes (FEV_1_ and FVC % predicted and FEV_1_/FVC) followed a normal distribution and results are expressed as mean and standard deviation (SD). Methacholine dose-response slope (MDRS) distribution was normalised using the transformation 100/(MDRS+10) = tMDRS[27,30]. The levels of specific IgE were subject to a log_e_-transformation prior to analysis; skin prick tests WMD were used as raw data. Tobacco smoking and allergen exposure data followed a log_e_-distribution.

The correlation between SPT-WMD and sIgE levels for each allergen was assessed using Spearman's rho test. The relationship between quantitative sensitisation and outcome measures was analyzed using linear regression and General Linear Model Univariate ANOVA, with a p-value of 0.05 considered as significant. Regression slope, R^2^ (as measure of effect direction and size, respectively) and 95% confidence intervals (95% CI) were estimated using linear regression models. General Linear Model multivariate ANCOVA analyses were performed, including all the factors identified as significant in the univariate analysis. Fitted predicted value curves according to the level of specific IgE/size of SPT-WMD were plotted using the results from the regression analysis. Statistical analysis was carried out using SPSS 15.0 (SPSS Inc., Chicago, IN, USA).

## Results

### Participants

We contacted 1446 parents of the children recruited in the Manchester Asthma and Allergy Study (population-based birth cohort representative of the general population). Of these, 178 declined the invitation and 99 did not respond. Of the 1169 subjects who expressed an interest, 831 (71.1%) signed informed consent, of whom 800 were included in the analysis (12 with insufficient data and 19 non-Caucasians were excluded). We then enriched the study population with 183 asthmatic patients, with the analysed sample including 983 Caucasian subjects (416 [42.3%] male; mean age 48.3 years, range 19.8-72.9). Demographic characteristics of the study population are presented in Table 1. Skin tests were performed on 730 subjects (74.3%) and 748 (76.1%) provided blood sample for IgE measurement; 680 subjects had both skin tests and IgE data. We found no significant differences between the subjects with and without skin tests and IgE data in any of the outcomes studied. Spirometry was done by 792 subjects (80.6%) and 626 (63.7%) underwent a methacholine challenge, with 100 (16.0%) having AHR (MDRS>25). Of the 748 subjects with IgE data, 473 (63.2%) were atopic with raised specific IgE to one or more allergens. Table 2 presents details of atopic status considering both dichotomous and quantitative definitions. Table 3 presents the correlation between SPT-WMD and sIgE levels for each allergen.

**Table 1:** Characteristics of the 983 subjects included in the study

Variable			Total (n)*	% of Total Subjects
**Age (y)**	**Mean**	**(Range)**		
	48.3	(19.8-72.9)	983	100.0
**Gender - male**	**n**	**(%)**		
	
	416	(42.3)	983	100.0
**BMI (%)**	**Mean**	**(SD)**		
	
	27.2	5.1	769	78.2
**Asthma**	**n**	**(%)**		
**Current Asthma**	312	(31.74)	983	100.0
**Airway Hyperresponsiveness (AHR)**	**n**	**(%)**		
	
**AHR-MDRS>25**	100	(15.97)	626	63.7
	**GM**	**(Range)**		
	
**MDRS**	8.229	(0.0-17592.5)	626	63.7
**Current Asthma with AHR**	**n**	**(%)**		
	
**Current Asthma + AHR-MDRS>25**	64	(10.22)	626	63.7
**Lung Function**	**Mean**	**(SD)**		
	
**FVC % predicted**	111.86	(16.01)	790	80.4
**FEV_1_ % predicted**	101.20	(18.41)	792	80.6
**FEV_1_/FVC**	75.84	(8.71)	790	80.4
**Atopy**	**n**	**(%)**		
	
**SPT**	402	55.07	730	74.3
**sIgE**	473	63.24	748	76.1
**Smoking**	**n**	**(%)**		
	
**Ever smokers**	396	(40.28)	983	100.0
**Current smokers**	114	(11.60)	983	100.0
	**GM**	**(Range)**		
	
**Smoke-pack-years**	0.0006	(0.0 -104.59)	981	99.8
**Pet ownership**	**n**	**(%)**		
	
**Cat owner (current)**	171	21.22	806	81.99
**Dog owner (current)**	142	17.62	806	81.99
**Cat or dog owner (current)**	277	34.37	806	81.99
	
**Indoor allergen exposure **(μg/g)	**GM**	**(Range)**		
**Mite (Der p 1)**	2.59	(0.20-188.67)	953	96.95
**Cat (Fel d 1)**	1.42	(0.04-5377.61)	951	96.75
**Dog (Can f 1)**	0.96	0.20-3361.02)	948	96.44

*Total number of patients with available data; BMI - Body mass index; AHR - Airway Hyperresponsiveness; MDRS - methacholine dose-response slope; SPT - skin prick tests; sIgE - serum specific Immunoglobulin E; GM - Geometric mean.

Notes: Current Asthma - Physician-diagnosed asthma with asthma symptoms and/or use of asthma medication in the last 12 months[26]**; f**or MDRS, GM calculated from tMDRS(= 100/(MDRS+10)[27,30]; for smoke-pack-years and indoor allergen exposure, GM calculated from log_e_ transformation.

**Table 2:** Allergen sensitisation: Dichotomous definitions on SPT and sIgE, SPT wheal mean diameters (WMD, mm) and sIgE level (kU_A_/l)

	Dichotomous		Quantitative		Total (n)	% of Total Subjects
**SPT**	**n**	**(%)**	Median*	**(Range)**		

**Mite**	256	(35.07)	4.5	(0-19.5)	730	74.26
**Cat**	175	(23.97)	4.5	(0-20.0)	730	74.26
**Dog**	112	(15.34)	4.0	(0-16.5)	730	74.26
**Tree pollen mix**	84	(14.43)	5.0	(0-11.0)	582	59.21
**Grass pollen mix**	213	(29.18)	5.0	(0-23.0)	730	74.26
**Mould mix**	32	(4.40)	4.0	(0-12.0)	727	73.96
**Specific IgE**	**n**	**(%)**	**GM**	**(Range)**		

**Mite**	343	(45.86)	0.48	(0.05-160.77)	748	76.09
**Cat**	211	(28.21)	0.16	(0.05-146.94)	748	76.09
**Dog**	170	(22.73)	0.14	(0.05-100.48)	748	76.09
**Grass pollen mix**	336	(44.92)	2.18	(0.05-407.48)	748	76.09

*Median SPT-WMD among subjects with positive SPTs only (median in whole population = 0 for all allergens due to non-sensitised subjects); GM - Geometric mean; SPT - skin prick tests.

Note: for specific IgE, GM was calculated based on log_e_ transformation.

**Table 3:** Correlation between SPT-WMD (mm) and sIgE levels (kU_A_/l) for each allergen

Allergen	Spearman's rho	p value
**Mite**	0.734	3.63 × 10^-116^
**Cat**	0.696	1.36 × 10^-99^
**Dog**	0.548	1.85 × 10^-54^
**Grass pollen**	0.644	5.51 × 10^-81^

### Specific IgE, Skin Prick tests and Lung Function

Table 4 present the associates of the lung function parameters studied. When using IgE measurement to define subjects as atopic or not (*i.e.*, dichotomous definition), there was a strong association between atopy and poorer lung function (FEV_1_ and FVC % predicted, FEV_1_/FVC) and the same was seen when using SPT to define atopy (Table 4). This was also true when looking at specific sensitisation to a range of inhalant allergens on either IgE or SPT, with particularly strong associations with sensitisation to cat (e.g., for FEV_1_ % predicted on specific IgE p = 0.0007, on SPT p = 5.02 × 10^-7^) and dog (for FEV_1_ % predicted on SPT p = 4.16 × 10^-12^, for sIgE p = 8.57 × 10^-8^).

**Table 4:** Associates of FEV_1_ and FVC % predicted and FEV_1_/FVC (univariate analysis)

		**FEV_1_ % predicted**			**FVC % predicted**			**FEV_1_/FVC**		
			
**Associate**		**P**	**Slope**	**R^2^**	**P**	**Slope**	**R^2^**	**P**	**Slope**	**R^2^**
			
Atopy on SPT*		0.00001	-5.93	0.03	0.03	-2.61	0.007	5.39×10^-8^	-9.81	0.03
**Mite**	**Positive SPT**	0.00001	-6.27	0.03	0.01	-3.25	0.009	6.17×10^-7^	-9.76	0.03
	**SPT-WMD**	8.23×10^-8^	-1.40	0.04	0.002	-0.75	0.01	4.12×10^-9^	-2.11	0.05
**Cat**	**Positive SPT**	5.02×10^-7^	-7.69	0.03	0.02	-2.96	0.007	1.08×10^-7^	-13.61	0.05
	**SPT-WMD**	3.93×10^-10^	-1.63	0.05	0.0007	-0.81	0.02	2.80×10^-10^	-2.55	0.06
**Dog**	**Positive SPT**	4.16×10^-12^	-12.22	0.06	0.0001	-6.06	0.02	1.38×10^-13^	-18.21	0.07
	**SPT-WMD**	3.03×10^-15^	-2.49	0.08	1.70×10^-6^	-1.41	0.03	8.53×10^-16^	-3.68	0.09
**Tree**	**Positive SPT**	0.73	0.52	0.0002	0.51	0.97	0.0008	0.85	-1.36	0.0005
	**SPT-WMD**	0.93	-0.07	0.00001	0.70	0.02	0.0003	0.37	-0.51	0.002
**Grass**	**Positive SPT**	2.06×10^-6^	-6.78	0.03	0.004	-3.64	0.01	2.79×10^-7^	-9.40	0.03
	**SPT-WMD**	2.95×10^-9^	-1.04	0.05	0.0002	-0.59	0.02	9.62×10^-10^	-1.50	0.05
**Mould**	**Positive SPT**	0.22	-3.22	0.002	0.61	-1.10	0.0004	0.17	-8.17	0.005
	**SPT-WMD**	0.02	-1.53	0.007	0.24	-0.77	0.002	0.006	-2.60	0.01
**Atopy on sIgE^#^**		0.0007	-4.83	0.02	0.10	-2.03	0.004	0.0001	-7.22	0.02
**Mite**	**Positive sIgE**	0.03	-2.97	0.006	0.45	-0.89	0.0008	0.008	-5.31	0.01
	**sIgE level**	0.001	-0.88	0.01	0.21	-0.30	0.002	0.0004	-1.49	0.02
**Cat**	**Positive sIgE**	0.0007	-5.16	0.02	0.57	-0.75	0.0004	0.00006	-11.76	0.04
	**sIgE level**	0.0001	-1.37	0.02	0.28	-0.33	0.002	0.00003	-2.78	0.04
**Dog**	**Positive sIgE**	8.57×10^-8^	-8.70	0.04	0.02	-3.39	0.008	7.30×10^-8^	-16.05	0.06
	**sIgE level**	2.95×10^-8^	-2.29	0.04	0.004	-1.04	0.01	1.96×10^-7^	-3.87	0.06
**Grass**	**Positive sIgE**	0.04	-2.82	0.006	0.72	-0.42	0.0002	0.002	-5.83	0.01
	**sIgE level**	0.15	-0.38	0.003	0.82	-0.05	0.00007	0.05	-0.84	0.006
**Gender - Female**		0.09	2.27	0.004	1.00×10^-6^	5.66	0.03	0.0009	0.02	0.01
**Age (years)**		1.22×10^-13^	-0.62	0.07	3.29×10^-6^	-0.30	0.03	1.31×10^-31^	-0.005	0.16
**BMI (%)**		0.0008	-0.42	0.01	4.80×10^-9^	-0.64	0.04	0.36	0.001	0.001
**Current Asthma**		1.18×10^-41^	-17.46	0.21	3.05×10^-13^	-8.53	0.07	3.68×10^-43^	-0.08	0.21
**Cat ownership (current)**		0.32	-1.65	0.001	0.96	-0.12	2.54×10^-6^	0.10	-0.01	0.003
**Dog ownership (current)**		0.25	-1.99	0.002	0.68	-0.60	0.0002	0.14	-0.01	0.003
**Ln Der p 1 Exposure (μg/g)**		0.62	0.17	0.0003	0.33	0.29	0.001	0.68	-0.001	0.0002
**Ln Fel d 1 Exposure (μg/g)**		0.39	-0.22	0.0010	0.77	0.08	0.0001	0.01	-0.003	0.008
**Ln Can f 1 Exposure (μg/g)**		0.0001	-1.22	0.02	0.002	-0.87	0.01	0.003	-0.005	0.01
**Smoking (Ever)**		0.0008	-4.46	0.01	0.07	-2.08	0.004	0.0001	-0.02	0.02
**Ln Smoke-Pack-Years**		0.0002	-0.31	0.02	0.03	-0.16	0.006	0.0001	-0.002	0.02

*At least one positive skin prick test result; #At least one positive sIgE result; SPT-WMD - Skin prick test wheal mean diameter (mm); sIgE level (kU_A_/l). BMI - Body mass index; AHR - Airway Hyperresponsiveness; MDRS - methacholine dose-response slope; SPT - skin prick tests; sIgE - serum specific IgE; GM - Geometric mean. Current Asthma - Physician-diagnosed asthma with asthma symptoms and/or use of asthma medication in the last 12 months[26].

**Figure 1:**
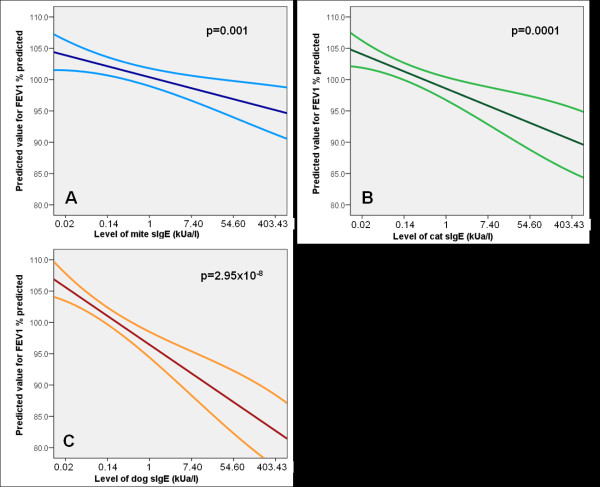
**Predicted value for FEV_1_ % predicted in relation to sensitization to mite (A), cat (B) and dog (C) - sIgE level (kU_A_/l), derived from the linear regression analysis (regression line with 95% CI)**.

**Figure 2:**
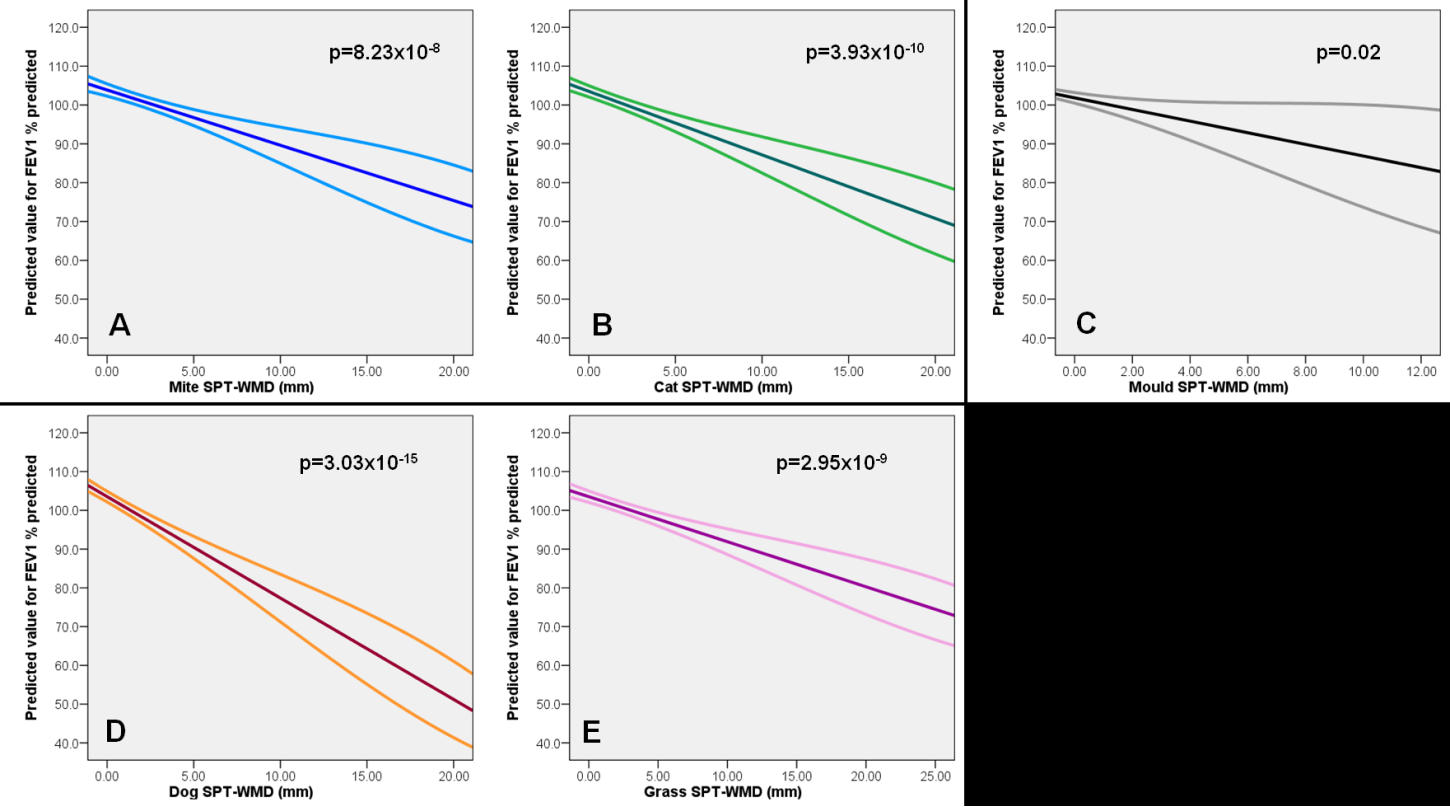
**Predicted value for FEV_1_ % predicted in relation to sensitization to mite (A), cat (B), moulds (C), dog (D) and grass pollen (E) - SPT-WMD (mm), derived from the linear regression analysis (regression line with 95% CI)**.

Using sIgE as a continuous variable in a linear regression analysis, we found that increasing levels of sIgE to mite, cat and dog were significantly associated with poorer level of FEV_1_ % predicted (for mite p = 0.001, cat p = 0.0001, dog p = 2.95 × 10^-8^, Table 4 and Figure 1); the same was also observed for FEV_1_/FVC, though only increasing levels of sIgE to dog were associated with poorer FVC % predicted (Table 4). Similar findings were observed when using SPT as continuous variable, with significantly poorer lung function with increasing size of SPT-WMD for inhalant allergens (Table 4). This is illustrated for FEV_1_ % predicted in Figure 2: mite p = 8.23 × 10^-8^, cat p = 3.93 × 10^-10^, dog p = 3.03 × 10^-15^, grass p = 2.95 × 10^-9^, mould p = 0.02.

When the levels of specific IgE for the individual allergens (mite, cat, dog, grass) were summed, the strength of this effect was slightly weakened or lost for FEV_1_ and FVC % predicted and FEV_1_/FVC (p = 0.01, p = 0.17 and p = 0.002, respectively). However, this was not the case when adding the sizes of SPT-WMD for the same allergens (FEV_1_ % predicted p = 1.89 × 10^-15^, FVC % predicted p = 3.20 × 10^-6^, FEV_1_/FVC p = 1.99 × 10^-16^).

### Specific IgE, Skin prick tests and Airway Hyperresponsiveness

**Figure 3:**
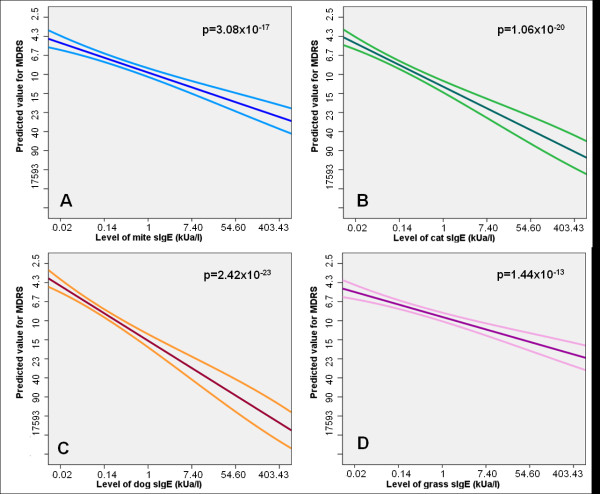
**Predicted value for MDRS in relation to sensitization to mite (A), cat (B), dog (C) and grass pollen (D) - sIgE level (kU_A_/l), derived from the linear regression analysis (regression line with 95% CI)**. *Note*: analysis done with tMDRS (= 100/(MDRS+10), with converted values shown in Y axis, hence inverted scale.

Associates of airway hyperresponsiveness (MDRS) from univariate regression analysis are shown in Table 5. Increased MDRS was strongly associated with atopy (on either SPT or sIgE, p ≤ 3.14 × 10-14) and with sensitisation to all inhalant allergens tested when using dichotomous definitions (Table 5). When analysing this using sensitisation as quantitative measure, we found that increasing levels of sIgE/increasing size of SPT-WMD were strongly associated with increasing MDRS (Table 5 and Figure 3, illustrative of these associations for sIgE level). When the levels of sIgE or SPT-WMD sizes to each individual allergen were summed, this association remained strongly significant (p = 1.63 × 10^-24^ for sum of sIgE or SPT-WMD).

**Table 5:** Associates of MDRS (univariate analysis)

		**tMDRS**		
	
**Associate**		**p**	**Slope**	**R^2^**
	
Atopy on SPT*		4.27×10^-11^	-1.43	0.07
**Mite**	**Positive SPT**	1.05×10^-9^	-1.43	0.06
	**SPT-WMD**	1.28×10^-12^	0.033	0.08
**Cat**	**Positive SPT**	4.31×10^-9^	-1.55	0.06
	**SPT-WMD**	3.09×10^-11^	-0.38	0.07
**Dog**	**Positive SPT**	4.30×10^-12^	-2.32	0.08
	**SPT-WMD**	3.95×10^-12^	-0.51	0.08
**Tree**	**Positive SPT**	0.0001	-1.19	0.03
	**SPT-WMD**	0.0001	-0.23	0.03
**Grass**	**Positive SPT**	1.27×10^-7^	-1.31	0.05
	**SPT-WMD**	5.80×10^-8^	-0.27	0.05
**Mould**	**Positive SPT**	0.003	-1.92	0.02
	**SPT-WMD**	0.002	-0.48	0.02
**Atopy on sIgE^#^**		3.14×10^-14^	-1.72	0.10
**Mite**	**Positive sIgE**	1.04×10^-11^	-1.54	0.08
	**sIgE level**	3.08×10^-17^	-0.39	0.12
**Cat**	**Positive sIgE**	4.83×10^-16^	-2.08	0.11
	**sIgE level**	4.83×10^-16^	-0.57	0.14
**Dog**	**Positive sIgE**	2.55×10^-21^	-2.59	0.15
	**sIgE level**	2.42×10^-23^	-0.72	0.16
**Grass**	**Positive sIgE**	7.73×10^-13^	-1.61	0.09
	**sIgE level**	1.44×10^-13^	-0.33	0.09
**Gender - Female**		1.07×10^-7^	-1.17	0.04
**Age (years)**		0.39	-0.04	0.001
**BMI (%)**		0.14	-0.03	0.004
**FEV_1_ % predicted**		2.55×10^-28^	0.08	0.18
**FVC % predicted**		0.001	0.03	0.02
**FEV_1_/FVC**		1.18×10^-27^	15.79	0.18
**Current Asthma**		3.30×10 ^-32^	-2.89	0.20
**Cat ownership (current)**		0.42	0.24	0.001
**Dog ownership (current)**		0.04	-0.61	0.007
**Ln Der p 1 Exposure (μg/g)**		0.77	0.02	0.0001
**Ln Fel d 1 Exposure (μg/g)**		0.26	0.06	0.002
**Ln Can f 1 Exposure (μg/g)**		0.02	-0.13	0.009
**Smoking (Ever)**		0.08	-0.40	0.005
**Ln Smoke-Pack-Years**		0.08	-0.03	0.005

*At least one positive skin prick test result; #At least one positive sIgE result; SPT-WMD - Skin prick test wheal mean diameter (mm); sIgE level (kU_A_/l)

BMI - Body mass index; MDRS - methacholine dose-response slope; SPT - skin prick tests; sIgE - serum specific IgE. Current Asthma - Physician-diagnosed asthma with asthma symptoms and/or use of asthma medication in the last 12 months[26].

Note: tMDRS = 100/(MDRS+10), thus negative slope means increasing MDRS

### Specific IgE antibodies, Skin Test Size and Lung Function and Airway Hyperresponsiveness amongst sensitised subjects

To further Investigate the relationship between quantitative atopy, lung function and AHR, we repeated the analysis amongst subjects who would be considered sensitised using standard definitions (e.g., sIgE>0.35 kUa/l, SPT-WMD 3 mm>negative control). Using these definitions, 473 subjects were assigned as sensitised based on sIgE and 402 based on SPT (Table 1). Even within the group of atopic individuals, increasing level of sIgE or SPT-WMD size to individual allergens was associated with significantly poorer lung function or increasing AHR (data shown for FEV_1_ % predicted and MDRS in Table 6).

**Table 6:** Quantification of IgE and SPT and FEV _1_ % predicted and tMDRS amongst sensitised subjects (univariate analysis)

	**FEV_1_ % predicted**			**tMDRS**		
		
	**p**	**Slope**	**R^2^**	**p**	**Slope**	**R^2^**
		
**SPT-WMD**						
**Mite**	0.001	-1.33	0.04	0.001	-0.19	0.03
**Cat**	2.26×10^-6^	-1.41	0.05	1.57×10^-5^	-0.27	0.06
**Dog**	3.69×10^-11^	-2.43	0.1	2.64×10^-6^	-0.37	0.07
**Tree**	0.84	0.07	0.0001	0.08	-0.12	0.01
**Grass**	3.77×10^-6^	-1.04	0.05	0.03	-0.11	0.01
**Mould**	0.17	-0.99	0.005	0.09	-0.29	0.009
**sIgE**						
**Mite**	0.09	-0.50	0.004	6.08×10^-6^	-0.28	0.06
**Cat**	0.01	-1.05	0.01	1.14×10^-10^	-0.46	0.12
**Dog**	9.33×10^-6^	-2.08	0.04	2.90×10^-12^	-0.59	0.13
**Grass**	0.35	0.34	0.002	0.02	-0.19	0.03

SPT-WMD - Skin prick test wheal mean diameter (mm); sIgE level (kU_A_/l)

SPT - skin prick tests; sIgE - serum specific IgE

Note: tMDRS = 100/(MDRS+10), thus negative slope means increasing MDRS

### Multivariate analysis

To assess the relative contribution of sIgE levels and size of SPT-WMD to lung function (FEV_1_ % predicted) and AHR (MDRS), we performed a multivariate ANCOVA, controlling for all the factors that we found associated with these outcomes in the univariate analyses, including current asthma, smoking habits and indoor allergen exposure. In these models (Table 7), increasing level of sIgE to dog remained a strong and highly significant independent associate of FEV_1_ and FVC % predicted and FEV_1_/FVC; the same was true for increasing level of sIgE/size of SPT-WMD to mite, which remained an independent associates of MDRS. Increasing level of sIgE to mite was an independent associate of FEV_1_/FVC. The significant independent associates and the size of their effect varied slightly depending on whether SPT or sIgE data were used.

**Table 7:** Independent associates of lung function and AHR (multivariate analysis)

FEV_1_ % predicted				
**Associate**	**Model 1 (SPT-WMD)**		**Model 2 (sIgE level)**	
		
	**Adj p**	**Adj R^2^**	**Adj p**	**Adj R^2^**
		
**Age**	5.82×10^-7^	0.04	5.91×10^-8^	0.04
**BMI**	0.09		0.48	
**Current Asthma**	1.64×10^-14^	0.08	3.34×10^-24^	0.14
**Ln Smoke-Pack-Years**	0.02	0.008	0.02	0.008
**Ln Can f 1 exposure**	0.06		0.07	
**Mite**	0.16		0. 69	
**Cat**	0.91		0.46	
**Dog**	0.003	0.01	0.10	
**Grass**	1.00		NI	
**Mould**	0.11		NA	

**FVC % predicted**				

**Associate**	**Model 1 (SPT-WMD)**		**Model 2 (sIgE level)**	
		
	**Adj p**	**Adj R^2^**	**Adj p**	**Adj R^2^**
		
**Age**	0.01	0.009	0.01	0.009
**BMI**	3.65×10^-5^	0.03	6.23×10^-5^	0.02
**Gender - female**	0.0003	0.02	0.001	0.02
**Current Asthma**	0.0002	0.02	2.91×10^-8^	0.04
**Ln Smoke-Pack-Years**	0.44		0.45	
**Ln Can f 1 exposure**	0.18		0.13	
**Mite**	0.49		NI	
**Cat**	0.91		NI	
**Dog**	0.047	0.006	0.56	
**Grass**	0.94		NI	

**FEV_1_/FVC**				

**Associate**	**Model 1 (SPT-WMD)**		**Model 2 (sIgE level)**	
		
	**Adj p**	**Adj R^2^**	**Adj p**	**Adj R^2^**
		
**Age**	9.99×10^-20^	0.12	2.51×10^-23^	0.13
**Gender - female**	0.02	0.008	0.01	0.009
**Current Asthma**	8.08×10^-15^	0.09	8.52×10^-21^	0.12
**Ln Smoke-Pack-Years**	0.004	0.01	0.004	0.01
**Ln Can f 1 exposure**	0.20		0.33	
**Ln Fel d 1 exposure**	0.27		0.24	
**Mite**	0.02	0.008	0.21	
**Cat**	0.84		0.72	
**Dog**	0.003	0.01	0.47	
**Grass**	0.96		NI	
**Mould**	0.23		NA	

**tMDRS**				

**Associate**	**Model 1 (SPT-WMD)**		**Model 2 (sIgE level)**	
		
	**Adj p**	**Adj R^2^**	**Adj p**	**Adj R^2^**
		
**Gender - female**	3.59×10^-13^	0.09	3.97×10^-13^	0.09
**Current Asthma**	3.82×10^-7^	0.05	0.0003	0.02
**FEV_1_ % predicted**	60.3×10^-9^	0.06	1.04×10^-8^	0.06
**FEV_1_/FVC**	0.001	0.02	2.05×10^-5^	0.03
**Ln Can f 1 exposure**	0.25		0.32	
**Mite**	0.0001	0.03	0.0004	0.02
**Cat**	0.36		0.45	
**Dog**	0.56		0.44	
**Grass**	0.20		0.007	0.01
**Mould**	0.30		NA	

Models using quantitative measures of sensitisation to inhalant allergens: either SPT-WMD in mm for Model 1 or level of sIgE in kU_A_/l for Model 2.

BMI - body mass index; tMDRS - transformed methacholine dose-response slope (tMDRS = 100/(MDRS+10); SPT - skin prick tests; sIgE - serum specific IgE.

Current Asthma - Physician-diagnosed asthma with asthma symptoms and/or use of asthma medication in the last 12 months[26].

NA - not available; NI - not included in model (no association in univariate analysis)

## Discussion

### Principal Findings

Our results confirm that sensitisation to inhalant allergens is an associate of lung function and airway hyperresponsiveness in adults. Furthermore, our data suggest that amongst adults there is a quantitative relationship between the level of allergen-specific IgE or the size of skin prick test reaction and the level of lung function and airway hyperresponsiveness, with decreasing lung function and increasing AHR with increasing level of specific IgE or skin test wheal size. We have demonstrated that the absolute level of specific IgE or the size of the skin test wheal diameter to mite and dog remain independent associates of lung function and airway hyperresponsiveness in our sample of adults in the UK after adjusting for potential confounding variables, including current asthma, smoking habits and indoor allergen exposure. In addition, we have extended this observation in demonstrating that the same associations remain within the group of subjects defined as atopic using standard definitions[6].

### Limitations and strengths

We were not able to obtain skin prick test data or specific IgE measurements for all subjects studied (74.3% had skin tests and 76.1% IgE). However, it is unlikely that this has influenced our results, since we found no significant differences between the subjects with and without these data in any of the outcomes studied. Moreover, the prevalence of allergic sensitisation among our subjects is similar to that of young adults in the UK[31], suggesting that our results are applicable to the general population.

As fewer subjects had skin prick testing data than measurements of specific IgE, fewer subjects were used in the analysis of the former, which may account for the small differences in the results (however, all of the trends remained the same). Similarly, some subjects in our study did not do lung function tests of had a methacholine challenge; however, there was no difference in atopy between the groups with and without these tests.

This was a cross-sectional study, and we cannot comment on the role of quantitative measures of allergen-specific serum IgE or skin prick tests on the change in lung function or AHR over time. This is an important question which needs to be addressed in cohort studies, which are unfortunately lacking in adults.

We acknowledge that the observed associations may differ between different ethnic groups. We decided to carry out our analysis amongst Caucasian participants, as the number of participants of other ethnic origins was too small to make any meaningful conclusions. We therefore wish to emphasise that our finding cannot be extrapolated to other ethnic groups, and further work is essential to address this important question.

We acknowledge that atopy is strongly associated with asthma, which could therefore be a confounder. However, by adjusting for asthma in the multiple regression models, we were able to take this into account and tease out independent associations between quantitative atopy, lung function and AHR. It is also possible that the association of quantitative atopy with lung function and AHR differs between asthmatics and healthy individuals. Our study population comprised a sample which is likely to be representative of the general population (800 parents of children enrolled in a population-based birth cohort), enriched by carefully phenotyped asthmatic subjects, giving us a total of 312 patients with current asthma. We enriched the study population with asthma cases to increase the range of lung function values and the number of subjects with AHR, and thus increase the power to detect associations. In addition, by enriching the study population with well-defined group of asthmatics, this design has increased our confidence that we could detect any difference between asthmatics and non asthmatics. In this population, we did not observe a significant difference between asthmatics and non-asthmatics with respect to the findings on the association between quantitative atopy and our outcomes of interest (i.e. lung function and AHR). However, this finding needs to be interpreted with great caution, as our study might not have sufficient power to detect such differences. This question needs to be addressed in a much larger study, using for example a matched case-control design.

### Interpretation

Many studies have identified allergic sensitisation (in particular to inhalant allergens) as a strong associate of lung function[1-8] and airway hyperresponsiveness[7-13], both in children and in adults, with reduced lung function and increased airway hyperresponsiveness in sensitised compared to non-sensitised individuals. However, as outlined previously, most such studies have considered sensitisation as either present or not[1-5,8-11]. A previous study by Burrows and colleagues[13] investigated the associates of AHR in children, looking at the sizes of skin test wheals when analysing atopy, and demonstrated that the sizes of the reactions to mite, cat, dog and *Aspergillus fumigatus *were independently correlated with airway hyperresponsiveness, particularly when summed. Another study by Nogalo et al.[12] looked specifically at the associations between airway hyperresponsiveness and level of specific mite specific IgE amongst mite-sensitised non-asthmatic children, and found that these were independently associated.

Our findings add to the above by demonstrating a quantitative rather than dichotomous relationship between atopy and the level of lung function and airway hyperresponsiveness in adults, when using both the size of skin prick tests and the level of specific IgE to inhalant allergens. In multivariate analyses adjusted for other relevant associates (such as asthma and smoking), we demonstrated significant independent associations for quantitative dog skin test and IgE levels and lung function, with quantitative mite IgE and skin test responses being associated with airway hyperresponsiveness. It is important to emphasise that the same quantitative relationships were demonstrated even when adults without allergic sensitisation were excluded from the analysis, confirming the importance of the quantitative approach.

Similar to our previous findings for lower respiratory symptoms and lung function in children[6], but in contrast to our findings for rhinitis and rhinoconjunctivitis[18], the associations between quantitative atopy and lung function and airway hyperresponsiveness remained strongly significant when the IgE levels/sizes of skin tests for individual allergens (mite, cat, dog and grass) were summed (with the single exception of the sum of sIgE levels and lung function parameters). Clearly, different clinical presentations of allergic airway disease are associated with different patterns of quantitative atopy, with the sum of inhalant allergens being important in childhood wheezing[6], grass pollen in seasonal and dust mite in perennial rhinitis[18], specific responses to dog in lung function and specific responses to dust mite in airway hyperresponsiveness. Dog allergen is readily inhaled into the lower airways, and may have an immediate impact on lung function. Dust mite allergens are carried on much larger particles and may contribute to the chronic inflammatory process best represented by airway hyperresponsiveness. The importance of dog and mite allergens in this study may also reflect the high concentrations of these allergens in UK homes. Our findings for lung function and airway hyperresponsiveness are consistent with recent studies demonstrating that quantification of IgE may be useful not only to diagnose allergic diseases in young children but to serve as a marker of persistence of wheeze[6] and severity of asthma[32,33].

## Conclusions

In our study population, we observed a quantitative relationship between IgE-mediated sensitisation and lung function or airway hyperresponsiveness in adults. Our results have important implications in clinical practice: the quantification of allergic sensitisation may offer more information to the clinician compared to the simple presence or absence of atopy.

## Abbrevations

**AHR**: Airway hyperresponsiveness; **BMI**: Body mass index; **FEV_1_**: Forced expiratory volume in one second; **FVC**: Forced vital capacity; **MDRS**: Methacholine dose-response slope; **sIgE**: specific serum immunoglobulin E; **SPT**: Skin prick tests; **SPT-WMD**: Skin prick test wheal mean diameter; **SPY**: Smoke pack years.

## Competing interests

The authors declare that they have no competing interests.

## Authors' contributions

SM participated in the design of the study, recruited and phenotyped the subjects, analysed and interpreted the data and drafted the manuscript. PM and JS phenotyped a subgroup of subjects. AS and AC conceived the study and its design, participated in its co-ordination and critically revised the manuscript. All authors read and approved the manuscript.

## References

[B1] Lowe LA Woodcock A Murray CS Morris J Simpson A Custovic A Lung function at age 3 years: effect of pet ownership and exposure to indoor allergens Arch Pediatr Adolesc Med 2004 158 996 1001 10.1001/archpedi.158.10.996 15466689

[B2] Lowe L Murray CS Custovic A Simpson BM Kissen PM Woodcock A Specific airway resistance in 3-year-old children: a prospective cohort study Lancet 2002 359 1904 1908 10.1016/S0140-6736(02)08781-0 12057553

[B3] Anthracopoulos MB Mantzouranis E Paliatsos AG Tzavelas G Lagona E Nicolaidou P Priftis KN Different effects of sensitization to mites and pollens on asthma symptoms and spirometric indices in children: a population-based cohort study Ann Allergy Asthma Immunol 2007 99 122 129 10.1016/S1081-1206(10)60635-7 17718099

[B4] Illi S von Mutius E Lau S Niggemann B Gruber C Wahn U Perennial allergen sensitisation early in life and chronic asthma in children: a birth cohort study Lancet 2006 368 763 770 10.1016/S0140-6736(06)69286-6 16935687

[B5] Hallberg J Anderson M Wickman M Svartengren M Factors in infancy and childhood related to reduced lung function in asthmatic children: a birth cohort study (BAMSE) Pediatr Pulmonol 2010 45 341 348 20306534 10.1002/ppul.21190

[B6] Simpson A Soderstrom L Ahlstedt S Murray CS Woodcock A Custovic A IgE antibody quantification and the probability of wheeze in preschool children J Allergy Clin Immunol 2005 116 744 749 10.1016/j.jaci.2005.06.032 16210045

[B7] Simpson A Tan VY Winn J Svensen M Bishop CM Heckerman DE Buchan I Custovic A Beyond atopy: multiple patterns of sensitization in relation to asthma in a birth cohort study Am J Respir Crit Care Med 2010 181 1200 1206 10.1164/rccm.200907-1101OC 20167852

[B8] Langley SJ Goldthorpe S Craven M Morris J Woodcock A Custovic A Exposure and sensitization to indoor allergens: association with lung function, bronchial reactivity, and exhaled nitric oxide measures in asthma J Allergy Clin Immunol 2003 112 362 368 10.1067/mai.2003.1654 12897743

[B9] Langley SJ Goldthorpe S Custovic A Woodcock A Relationship among pulmonary function, bronchial reactivity, and exhaled nitric oxide in a large group of asthmatic patients Ann Allergy Asthma Immunol 2003 91 398 404 10.1016/S1081-1206(10)61688-2 14582820

[B10] Fowler SJ Lipworth BJ Relationship of skin-prick reactivity to aeroallergens and hyperresponsiveness to challenges with methacholine and adenosine monophosphate Allergy 2003 58 46 52 10.1034/j.1398-9995.2003.23779.x 12580806

[B11] Craig TJ King TS Lemanske RF Jr Wechsler ME Icitovic N Zimmerman RR Jr Wasserman S Aeroallergen sensitization correlates with PC(20) and exhaled nitric oxide in subjects with mild-to-moderate asthma J Allergy Clin Immunol 2008 121 671 677 10.1016/j.jaci.2007.12.1153 18234311

[B12] Nogalo B Miric M Maloca I Turkalj M Plavec D Normal variation of bronchial reactivity in nonasthmatics is associated with the level of mite-specific IgE J Asthma 2008 45 273 277 10.1080/02770900701847084 18446590

[B13] Burrows B Sears MR Flannery EM Herbison GP Holdaway MD Relations of bronchial responsiveness to allergy skin test reactivity, lung function, respiratory symptoms, and diagnoses in thirteen-year-old New Zealand children J Allergy Clin Immunol 1995 95 548 556 10.1016/S0091-6749(95)70317-9 7852671

[B14] Gergen PJ Turkeltaub PC The association of individual allergen reactivity with respiratory disease in a national sample: data from the second National Health and Nutrition Examination Survey, 1976-80 (NHANES II) J Allergy Clin Immunol 1992 90 579 588 10.1016/0091-6749(92)90130-T 1401641

[B15] Sears MR Herbison GP Holdaway MD Hewitt CJ Flannery EM Silva PA The relative risks of sensitivity to grass pollen, house dust mite and cat dander in the development of childhood asthma Clin Exp Allergy 1989 19 419 424 10.1111/j.1365-2222.1989.tb02408.x 2758355

[B16] Sampson HA Update on food allergy J Allergy Clin Immunol 2004 113 805 819 10.1016/j.jaci.2004.03.014 15131561

[B17] Sporik R Hill DJ Hosking CS Specificity of allergen skin testing in predicting positive open food challenges to milk, egg and peanut in children Clin Exp Allergy 2000 30 1540 1546 11069561 10.1046/j.1365-2222.2000.00928.x

[B18] Marinho S Simpson A Soderstrom L Woodcock A Ahlstedt S Custovic A Quantification of atopy and the probability of rhinitis in preschool children: a population-based birth cohort study Allergy 2007 62 1379 1386 10.1111/j.1398-9995.2007.01502.x 17822449

[B19] Simpson BM Custovic A Simpson A Hallam CL Walsh D Marolia H Campbell J Woodcock A NAC Manchester Asthma and Allergy Study (NACMAAS): risk factors for asthma and allergic disorders in adults Clin Exp Allergy 2001 31 391 399 10.1046/j.1365-2222.2001.01050.x 11260150

[B20] Custovic A Simpson BM Murray CS Lowe L Woodcock A The National Asthma Campaign Manchester Asthma and Allergy Study Pediatr Allergy Immunol 2002 13 Suppl 15 32 37 12688622 10.1034/j.1399-3038.13.s.15.3.x

[B21] Janson C Anto J Burney P Chinn S de Marco R Heinrich J Jarvis D Kuenzli N Leynaert B Luczynska C The European Community Respiratory Health Survey: what are the main results so far? European Community Respiratory Health Survey II Eur Respir J 2001 18 598 611 10.1183/09031936.01.00205801 11589359

[B22] Pellegrino R Viegi G Brusasco V Crapo RO Burgos F Casaburi R Coates A van der Grinten CP Gustafsson P Hankinson J Interpretative strategies for lung function tests Eur Respir J 2005 26 948 968 10.1183/09031936.05.00035205 16264058

[B23] Miller MR Hankinson J Brusasco V Burgos F Casaburi R Coates A Crapo R Enright P van der Grinten CP Gustafsson P Standardisation of spirometry Eur Respir J 2005 26 319 338 10.1183/09031936.05.00034805 16055882

[B24] Crapo RO Casaburi R Coates AL Enright PL Hankinson JL Irvin CG MacIntyre NR McKay RT Wanger JS Anderson SD Guidelines for methacholine and exercise challenge testing-1999. This official statement of the American Thoracic Society was adopted by the ATS Board of Directors, July 1999 Am J Respir Crit Care Med 2000 161 309 329 10619836 10.1164/ajrccm.161.1.ats11-99

[B25] Simpson A Simpson B Custovic A Craven M Woodcock A Stringent environmental control in pregnancy and early life: the long-term effects on mite, cat and dog allergen Clin Exp Allergy 2003 33 1183 1189 10.1046/j.1365-2745.2003.01651.x 12956752

[B26] de Marco R Marcon A Jarvis D Accordini S Almar E Bugiani M Carolei A Cazzoletti L Corsico A Gislason D Prognostic factors of asthma severity: A 9-year international prospective cohort study J Allergy Clin Immunol 2006 117 1249 1256 10.1016/j.jaci.2006.03.019 16750983

[B27] Chinn S Burney P Jarvis D Luczynska C Variation in bronchial responsiveness in the European Community Respiratory Health Survey (ECRHS) Eur Respir J 1997 10 2495 2501 10.1183/09031936.97.10112495 9426085

[B28] O'Connor G Sparrow D Taylor D Segal M Weiss S Analysis of dose-response curves to methacholine. An approach suitable for population studies Am Rev Respir Dis 1987 136 1412 1417 10.1164/ajrccm/136.6.1412 3318599

[B29] Simpson A Simpson B Custovic A Craven M Woodcock A Stringent environmental control in pregnancy and early life: the long-term effects on mite, cat and dog allergen Clin Exp Allergy 2003 33 1183 1189 10.1046/j.1365-2745.2003.01651.x 12956752

[B30] Chinn S Choosing a transformation J Appl Stat 1996 23 395 404 10.1080/02664769624134

[B31] Broadfield E McKeever TM Scrivener S Venn A Lewis SA Britton J Increase in the prevalence of allergen skin sensitization in successive birth cohorts J Allergy Clin Immunol 2002 109 969 974 10.1067/mai.2002.124772 12063526

[B32] Wickman M Ahlstedt S Lilja G van Hage HM Quantification of IgE antibodies simplifies the classification of allergic diseases in 4-year-old children. A report from the prospective birth cohort study--BAMSE Pediatr Allergy Immunol 2003 14 441 447 10.1046/j.0905-6157.2003.00079.x 14675470

[B33] Wickman M Experience with quantitative IgE antibody analysis in relation to allergic disease within the BAMSE birth cohort--towards an improved diagnostic process Allergy 2004 59 Suppl 78 30 31 15245354 10.1111/j.1398-9995.2004.00572.x

